# Effects of Various Natural Diets on Gonad Development, Roe Quality, and Intestinal Microbiota of the Purple Sea Urchin (*Heliocidaris crassispina*)

**DOI:** 10.1155/anu/3196037

**Published:** 2025-02-28

**Authors:** Zhiyuan Wang, Guodong Wang, Hui Ge, Lili Zhang

**Affiliations:** ^1^State Key Laboratory of Mariculture Breeding, Fisheries College of Jimei University, Xiamen 361021, China; ^2^Key Laboratory of Healthy Mariculture for the East China Sea, Ministry of Agriculture and Rural Affairs, Fisheries College of Jimei University, Xiamen 361021, China; ^3^Fisheries Research Institute of Fujian, 7 Shanhai Road, Huli, Xiamen 361000, China

**Keywords:** *Heliocidaris crassispina*, gonadal development, intestinal microbiota, roe quality

## Abstract

The study analyzed the impact of different diets on the gonadal development of sea urchin *Heliocidaris crassispina*. Kelp (*Laminaria japonica*), corn (*Zea mays*), carrots (*Daucus carota*), and sweet potatoes (*Ipomaea batatas*) were used to continuously feed adults of *H. crassispina* for 50 days. Results indicated that sea urchins fed with kelp had the highest weight gain rate (*p* < 0.05), followed by those fed with sweet potato, no significant difference in weight gain rate was observed between other diets and no feeding groups (*p*  > 0.05). *H. crassispin* fed with corn had a significantly enhancing GSI (gonadsomatic index) (*p* < 0.05), followed by those fed kelp, and no significant difference between the other diet groups, but their weight gain rate was higher than that of the no feeding group (*p*  > 0.05). While there was no significant difference in shell diameter and height in any diets (*p*  > 0.05). Sweet potatoes and corn significantly improved the redness (a^*∗*^) and yellowness (b^*∗*^) of the gonads (*p* < 0.05). Kelp group and corn group had excellent performance in amino acid composition, containing higher levels of umami and sweet amino acids than other groups (*p* < 0.05). Fatty acid analysis showed higher contents of eicosapentaenoic acid (EPA), arachidonic acid (ARA), linolenic acid, and linoleic acid in kelp and corn group. The types of diets significantly affected the microbial diversity of the digestive tract, with kelp enhancing microbial community diversity, and diets of corn and sweet potatoes increasing the abundance of *Lactococcus*. In conclusion, kelp was an excellent feed for *H. crassispina*, and corn as a preferred alternative diet not only improved the GSI but also optimized the gonad color and increased the content of amino acids and fatty acids.

## 1. Introduction

The purple sea urchin (*Heliocidaris crassispina*), belonging to the phylum Echinodermata, class Echinoidea, order Camarodonta, and family Echinometridae, is renowned for its delicious gonads, commonly known as sea urchin roe, which hold high nutritional and medicinal value [1]. In China, the main cultured sea urchin are two cold-water species, *Strongylocentrotus intermedius* and *Strongylocentrotus nudus*, which cannot tolerate high temperatures. Instead, the purple sea urchin *H. crassispina* exhibits a high-temperature tolerance and presents the potential for aquaculture. Its roe supply is seasonally different from that of other cold-water sea urchins, which facilitates a staggered market. The adult cultivation of *H. crassispina* primarily relies on feeding large algae such as *Laminaria japonica* and *Gracilaria* [2], but these large algae cannot tolerate high temperatures of summer. The situation is that the seasonal supply of primary diets like large algae limits the development of the sea urchin farming industry [3]. Therefore, finding effective alternative diets available throughout the year is crucial not only for ensuring a stable food supply but also for improving the color and taste of the gonads.

Diet is a crucial factor affecting the development of sea urchin gonads [4, 5]. Currently, the development of sea urchin resources is at a low level [3, 6, 7]. The market price of sea urchin largely depends on the quality of the gonads, including parameters such as GW, gonadsomatic index (GSI), color, and moisture content [8]. Nutrients found in the gonads, such as proteins, amino acids, unsaturated fatty acids, carotenoids, and carbohydrates, are crucial indicators of gonad quality [9, 10]. Among these, amino acids not only serve important physiological functions but also play a significant role in flavor perception [11–14]. Fatty acids, as the primary nutrients in sea urchin gonads, offer potential benefits for cardiovascular disease prevention and promote embryonic and larval development in sea urchins [15, 16]. Current research has identified various nutritional needs for sea urchins, such as proteins, fats, n-3 long-chain polyunsaturated fatty acids (LC-PUFAs), and arachidonic acid (ARA) [17–19]. Enhancing the color and flavor of gonads has emerged as a new direction for the development of sea urchin aquaculture. Studies have shown that feeding sea urchins with different natural diets could significantly improve the color and sweetness of their gonads [7, 20].

In 1998, an investigation using corn (*Zea mays*) as an alternative feed to algae in sea urchin cultivation showed that corn not only promotes sea urchin growth but also significantly enhances the food conversion rate [21]. Another study demonstrated that feeding sea urchin *Paracentrotus lividus* solely with corn and spinach for 3 weeks could increase the gonad index by 135% [22]. Corn, as a primary staple crop in China, is readily available. Sweet potatoes (*Ipomoea batatas*), rich in lysine and methionine, which are crucial for the unique flavor of sea urchin gonads, also represent potential feed options [23]. Carrots with a high level of carotenoids, serve as another alternative feed since the carotenoid content directly affects the color of sea urchin gonads, thereby improving their quality [24, 25]. The aforementioned studies suggest that feeding sea urchins with natural diets such as corn, sweet potatoes, and carrots might not only shorten the cultivation period but also yield higher quality products. Moreover, intestinal microorganisms play a crucial role in the nutrient absorption and metabolic processes of sea urchins [26, 27]. The intestinal microbiota, a dominant component of the intestinal microecology, not only serves as one of the food sources for the host but also supplies a variety of essential nutrients for growth [28]. It can also be used to assess the sea urchin's ability to colonize microbes based on the intestinal content, providing insights into the nutritional metabolic levels.

Therefore, this study explored the effects of different diets on the growth, gonad development, roe quality, and intestinal microbiota of *H crassispina*, aiming to rapidly develop market-ready gonads and enhance roe quality by feeding nutritionally rich natural foods.

## 2. Materials and Methods

### 2.1. Animal Ethics

Rare or protected animals were not included in the experiments of this study. This study was approved by the Animal Care Advisory Committee of Jimei University (Approval No. 2019–0906-003, September 6, 2019).

### 2.2. Management of Sea Urchin Cultivation

Adult sea urchins (*N* = 500), with test diameters ranging from 55 to 70 mm and body weights between 40 and 70 g, were collected in August 2023 from the coastal areas of Pingtan, Fujian Province. Upon arrival at the laboratory, a subset of the sea urchins was randomly selected for weighing to establish initial baseline data for body weight and test diameter (*T* = 0). The specimens were then subjected to starvation for a week to synchronize their GSI. Sea urchins were randomly assigned to five groups for evaluating the effectiveness of several natural diets. They were Group M1 fed kelp, which was the most common in the daily diet of sea urchins, Group M2 diet consisted of fresh corn kernel, Group M3 20 mm thick carrot slices, Group M4 cut sweet potatoes 20 mm thick, and Group M5 no feeding group. All groups were cultured lasting 50 days. Every group had three replications and each replication had 10 sea urchin. Before each feeding, fecal pellets and unconsumed food were removed from each basket. Water was changed every 2 days, replacing one-third of the total volume. Water temperature was maintained between 26°C and 28°C, salinity at 32–33.5, pH between 7.97 and 8.08, with a light intensity of 2000 lux, and continuous aeration was provided.

### 2.3. Sampling and Measurement

At the end of the experiment, sea urchin shell diameter and shell height were measured using a caliper (DELIXI, Germany). The wet weight of the sea urchins was measured using an electronic analytical balance (Sartorius, Germany). After dissection, the GW was measured. The extracted sea urchin gonads were placed into zip-lock bags and squeezed to ensure the surface was flat and free of air bubbles. Gonad color was measured using a spectrophotometer (CM-700d, Konica Minolta, Japan) in accordance with the CIELAB standard; each sample was measured three times and the average was taken. Color difference (*Δ*E) between the measured color and standard colors (orange-yellow and bright yellow) was calculated according to the method of McBride [29]. Subsequently, samples of intestines, intestinal contents, and gonads were stored at −80°C.

### 2.4. Histologically

The histological analysis of gonadal tissues was conducted following the reference method provided by Santos et al. [30]. Collected sea urchin gonads were fixed in 4% paraformaldehyde for over 24 h to ensure the integrity of the tissue structure. The fixed samples were then dehydrated through a graded series of alcohol solutions, sequentially in 70%, 80%, 90%, and 100% ethanol. The samples were cleared twice in xylene (each for 30 min), followed by paraffin embedding. The embedded blocks were sectioned into thin slices of 5 μm thickness using a microtome. The paraffin sections were stained with hematoxylin and eosin (H&E) and observed under a microscope. Subsequently, the tissue structure characteristics were analyzed according to the gonadal development stages categorized by Byrne [31].

### 2.5. Nutritional Component Analysis

#### 2.5.1. Amino Acid Determination

Under nitrogen storage conditions, amino acids in the samples were released using an alkaline hydrolysis method to prevent oxidation. Samples are appropriately diluted based on the anticipated amino acid concentrations to fit the detection range of the *amino acid analyzer*. Processed samples were then conveyed to an amino acid analyzer through an automatic sampling system and separated on a chromatographic column. This process employed ion-exchange chromatography, utilizing pH and ionic strength gradients for elution to effectively separate the amino acids. After separation, amino acids were detected and analyzed both qualitatively and quantitatively by comparing their chromatograms with those of standard samples.

#### 2.5.2. Fatty Acid Determination

A mixed standard solution containing 51 types of fatty acid methyl esters was prepared, and metabolites were extracted from samples using the method described by Hoving et al. [32]. Fatty acid methyl ester solutions were injected into the chromatograph using a syringe through a split injection method, with an injection volume of 1 µL and a split ratio of 8:1. The injector temperature was set to 250°C, ion source temperature at 300°C, and transfer line temperature at 280°C. Helium was used as the carrier gas with a flow rate of 0.63 mL/min. Fatty acids were separated in the chromatographic column based on their chain length and saturation differences. Finally, quantitative analysis of fatty acids was performed by comparing the peak area ratios of samples to those of standard substances.

### 2.6. Intestinal Microbiome Analysis

#### 2.6.1. DNA Extraction and Quality Assessment

Intestinal wall and intestinal contents samples were grouped according to sea urchins on different diets. A1–E1 is the intestinal wall of sea urchin, and A2–E2 is the intestinal contents. Genomic DNA from the intestine and contents was extracted using the E. Z. N. A. Soil DNA Kit (Omega Bio-tek, Inc., USA). The quality and concentration of DNA were measured using a Nanodrop 2000 (ThermoFisher Scientific, Inc., USA). DNA samples were stored at −20°C for subsequent experiments.

#### 2.6.2. PCR Amplification

The V3–V4 region of the bacterial 16S rRNA gene was amplified using universal primers 338F (5′-ACTCCTACGGGAGGCAGCAG-3′) and 806R (5′-GGACTACHVGGGTWTCTAAT-3′). An 8 bp barcode sequence was added to the 5′ end of both the forward and reverse primers to differentiate between samples. The barcoded universal primers are synthesized and PCR amplification is conducted on an ABI 9700 PCR system (Applied Biosystems, Inc., USA). Alpha diversity analysis is performed using Mothur software. Principal component analysis (PCA) and heatmap analysis to assess the differences in bacterial community structure across different samples and groups are conducted using R software.

### 2.7. Calculations Statistical Analysis

The calculation formulas for indicators comprise the following:

  $$\begin{array}{c} \text{Weight growth rate }\left(\text{WGR},\%\right)=\frac{\left(\text{FBW}-\text{IBW}\right)}{\text{ IBW}\times 100}, \end{array}$$

  $$\begin{array}{c} \text{Shell diameter growth rate }\left(\text{SDGI},\%\right)=\frac{\left(\text{FBSD}-\text{IBSD}\right)}{\text{ IBSD}\times 100}, \end{array}$$

  $$\begin{array}{c} \text{Shell high growth rate }\left(\text{SHGI},\%\right)=\frac{\left(\text{FBSH}-\text{IBSH}\right)}{\text{ IBSH}\times 100}, \end{array}$$

  $$\begin{array}{c} \text{Gonadosomatic index }\left(\text{GSI},\%\right)=\frac{\text{GW}}{\text{ FBW}\times 100}. \end{array}$$

IBW and FBW represent the initial and final body weight in each sample, respectively. IBSD and FBSD represent the initial and final shell diameters of each sample, respectively. IBSH and FBSH represent the initial and final shell high for each sample. GW represents the individual gonad weight of sea urchin at each sampling time.

The homogeneity test of variance was conducted by SPSS 20.0 software, and the differences between different diets were compared by one-way analysis of variance and Duncan multivariate range test. A *p*-value of less than 0.05 was considered statistically significant. Statistical results are presented as mean ± standard error (mean ± SE).

## 3. Results

### 3.1. Effects of Different Diets on Growth and Gonad Development of *H. crassispina*

#### 3.1.1. Growth

**Figure 1:**
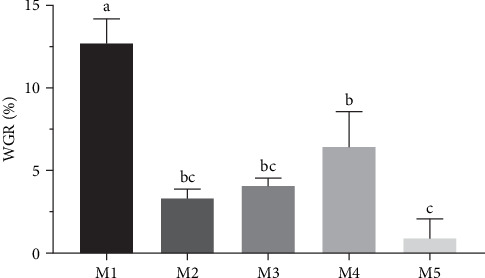
Effects of different diets on the WGR of *H. crassispina*. *Note*: Group M1 fed kelp. Group M2 corn. Group M3 carrot. Group M4 sweet potatoes. Group M5 no feeding. Values with superscript indicate significant differences between different groups in the inter group at *p* < 0.05. WGR, weight growth rate.

Significant differences were observed in the weight gain rates of sea urchins fed different diets (*p* < 0.05) (Figure 1). The kelp group exhibited the highest weight gain rate (*p* < 0.05), followed by sweet potato group (*p* < 0.05). No significant differences were found between corn group and carrot group (*p* > 0.05), while no feeding group had the lowest rate of weight gain (*p* < 0.05). Shell diameter and shell high growth rate were not significantly different among all groups (*p* > 0.05) (Table 1).

#### 3.1.2. Gonad Yield

**Figure 2:**
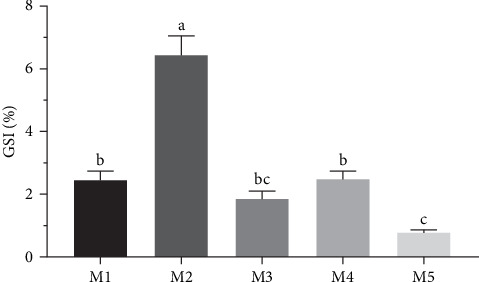
Effects of different diets on GSI of *H. crassispina*. *Note*: Group M1 fed kelp. Group M2 corn. Group M3 carrot. Group M4 sweet potatoes. Group M5 no feeding. Values with superscript indicate significant differences between different groups in the inter group at *p* < 0.05. GSI, gonadosomatic index.

The gonadal index of purple sea urchins reared on different diets is presented (Figure 2). Corn group exhibited the highest gonadal index, significantly outperforming all other groups (*p* < 0.05). There were no significant differences between the kelp group, sweet potato group, and carrot group (*p* > 0.05), but all were significantly higher than no feeding group (*p* < 0.05).

#### 3.1.3. Histological Observations of Gonadal Development

**Figure 3:**
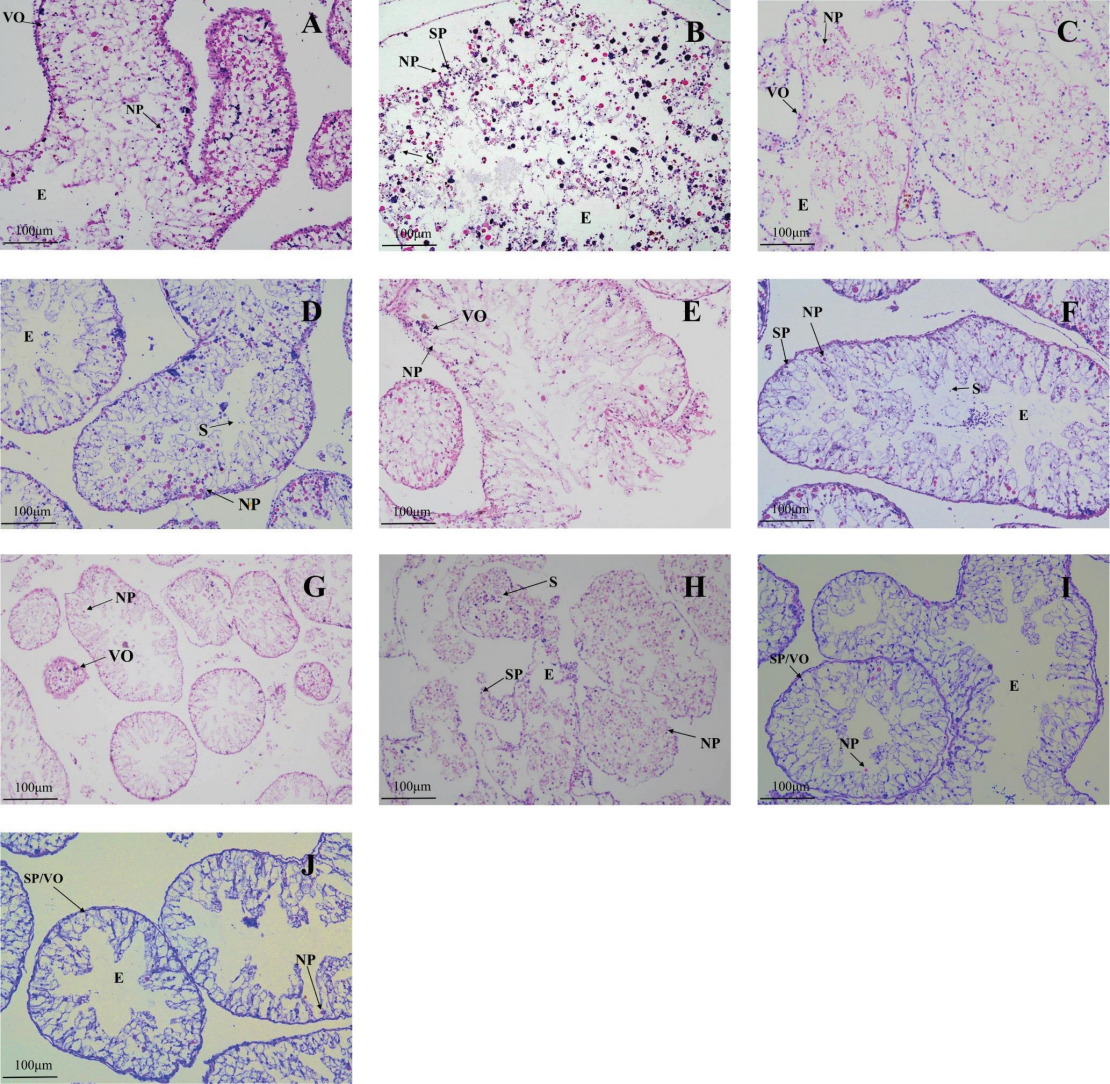
The gonadal tissues of *H. crassispina* fed with different diets for 50 days. (A, B) Corn, female, and male; (C, D) kelp, female, and male; (E, F) sweet potato, female, and male; (G, H) carrot, female, and male; (I, J) no feeding, female, and male. NP, nutrient phagocyte; SP, spermatocytes; S, sperm; VO, oocytes; O, ovum; E, cavity.

The gonadal development of both male and female sea urchins in all groups was immature (Figure 3). In female sea urchins, corn group (Figure 3A) showed a relatively faster development of gonads, with a noticeably higher number of oocytes closely packed together, yet without forming mature gametes, indicating a stage III of development. The kelp and sweet potato groups (Figure 3C,E) displayed fewer early-stage oocytes, just entering stage III. In male sea urchins, corn group (Figure 3B) had a higher sperm count, nearing the end of stage III; whereas other groups such as the male sweet potato and carrot groups (Figure 3F,H) had noticeably fewer sperms, likely at the late stage II or early stage III. No feeding group (Figure 3I,J) exhibited the slowest gonadal development among all, with all individuals remaining at stage I.

#### 3.1.4. Gonadal Coloration Analysis

**Figure 4:**
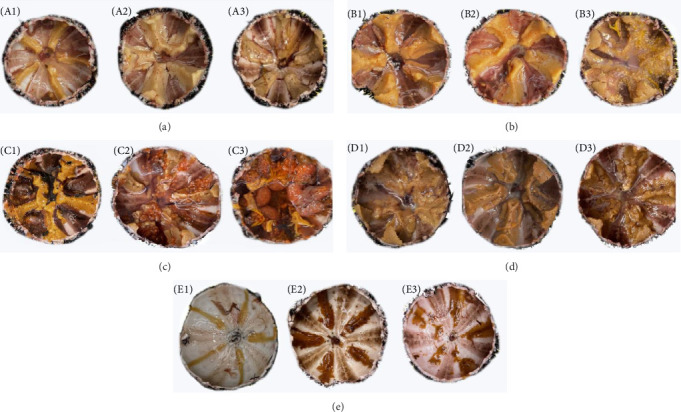
Color of gonads of *H. crassispina* fed on different diets. (A; A1–A3) was the gonads of sea urchins fed in kelp group, (B; B1–B3) was corn fed group, (C; C1–C3) was carrot fed group, (D; D1–D3) was sweet potato fed group, and (E; E1–E3) was no feeding group.

The luminance values of the gonads in kelp and corn group were significantly higher than those in carrot group and sweet potato groups and were also significantly higher than those in no feeding group (*p* < 0.05). Conversely, the a*⁣*^*∗*^ value (redness) was significantly higher in carrot and sweet potato groups compared to other groups (*p* < 0.05). Corn group exhibited a significantly higher b*⁣*^*∗*^ value (yellowness) in the gonads compared to other groups, with no feeding group having the lowest (*p* < 0.05). In no feeding group, the highest values of *Δ*E_1_ and *Δ*E_2_ were observed in the gonads. There was no significant difference between kelp, corn, and sweet potato groups (*p* > 0.05), but these values were significantly lower than those in carrot group (*p* < 0.05) (Table 2 and Figure 4).

### 3.2. Impact of Different Diets on the Nutritional Gonads in *H. crassispina*

#### 3.2.1. Amino Acid Composition

**Figure 5:**
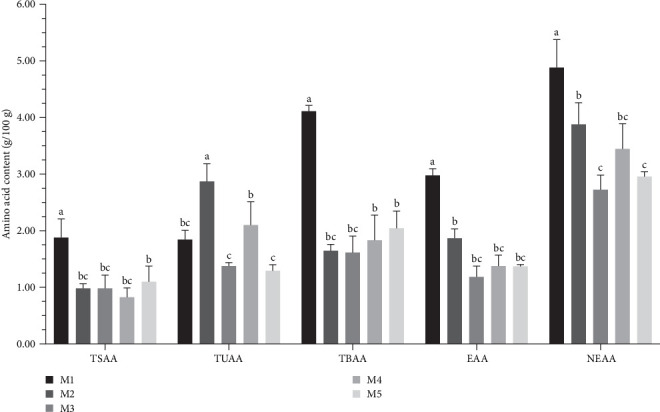
Gonad amino acid composition of *H. crassispina* fed on different diets (WM basis). *Note*: Group M1 fed kelp. Group M2 corn. Group M3 carrot. Group M4 sweet potatoes. Group M5 no feeding. Umami amino acids (TSAA): aspartic acid, glutamate. Sweet amino acids (TUAA): threonine, serine, glycine, alanine, and proline. Bitter amino acids (TBAA): cysteine, valine, methionine, isoleucine, leucine, tyrosine, phenylalanine, lysine, histidine, and arginine. Values with superscript indicate significant differences between different groups in the inter group at *p* < 0.05.

The kelp group led in the content of total amino acids (TAAs), essential amino acids (EAAs), nonessential amino acids (NEAAs), tasty amino acids (TSAAs), and tasty and bitter amino acids (TBAAs). Specifically, the TAA content in the kelp group was 7.822 g/100 g of gonads, which was second only to no feeding group and significantly higher than corn group, carrot group, and sweet potato group (*p* < 0.05). In terms of EAA and NEAA content, kelp group had 2.999 g/100 g and 4.823 g/100 g, respectively, significantly surpassing the other experimental groups and no feeding group (*p* < 0.05). Regarding specific taste-related amino acid categories, kelp group had the richest content of TBAAs (1.823 g/100 g and 4.11 g/100 g, respectively), with significant differences compared to other groups (*p* < 0.05). However, corn group had the highest content of sweet-tasting amino acids (TUAAs), significantly more than all other groups (*p* < 0.05), followed closely by sweet potato group, with carrot group having the lowest content (Table 3 and Figure 5).

#### 3.2.2. Fatty Acid Composition

**Figure 6:**
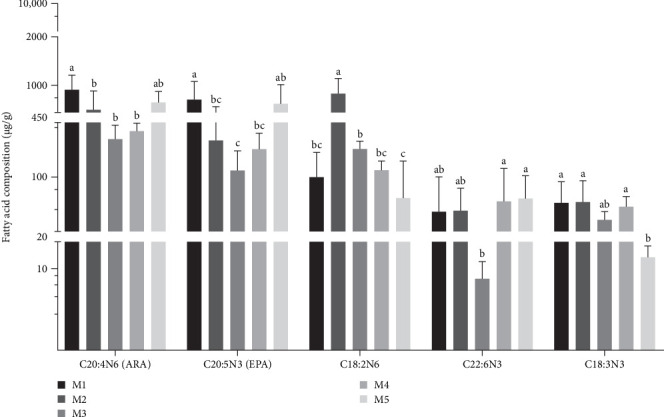
Content of special fatty acids in gonads of *H. crassispina* fed with different dites (WM basis). *Note*: Values with superscript indicate significant differences between different groups in the inter group at *p* < 0.05.

In terms of specific fatty acids, the content of eicosapentaenoic acid (EPA) in the kelp group was significantly higher than in other groups (*p* < 0.05). Docosahexaenoic acid (DHA) had the highest content in no feeding group, although there was no significant difference compared to sweet potato group, while carrot group's content was significantly lower than in other groups (*p* < 0.05). ARA content was significantly higher in the kelp group compared to all other groups, and no feeding group's content was significantly higher than in corn group, carrot group, and sweet potato groups (*p* < 0.05). For saturated fatty acids (SFAs) and monounsaturated fatty acids (MUFAs), the total content in sweet potato group and corn groups was significantly higher than those in other groups (*p* < 0.05), with no feeding group having the lowest content. Concerning omega-3 (n-3) PUFAs, the kelp group and no feeding group had higher contents, significantly surpassing corn group, carrot group, and sweet potato group (*p* < 0.05). The total content of omega-6 (n-6) PUFAs was highest in corn group, while it was lower in kelp group and no feeding group but significantly higher than in carrot group and sweet potato group (*p* < 0.05). The ratio of n-3 to n-6 PUFA was most balanced in no feeding group, significantly higher than that in the kelp group and sweet potato group (*p* < 0.05), while corn group and carrot group had the lowest ratios (Table 4 and Figure 6).

### 3.3. Intestinal Microbiota

#### 3.3.1. High-Throughput Sequencing Analysis

In the 16S rRNA sequencing of the intestinal microbiota of fed different diets from the purple sea urchin, a total of 2,185,919 high-quality sequences were obtained, accounting for 96.56% of the data. Clustering yielded 856 operational taxonomic units (OTUs), with 855 remaining after rarefaction. There were no contents in the intestine of the sea urchins in no feeding group, so E2 samples were not sequenced.

#### 3.3.2. Microbial Diversity

##### 3.3.2.1. Alpha Diversity Analysis

**Figure 7:**
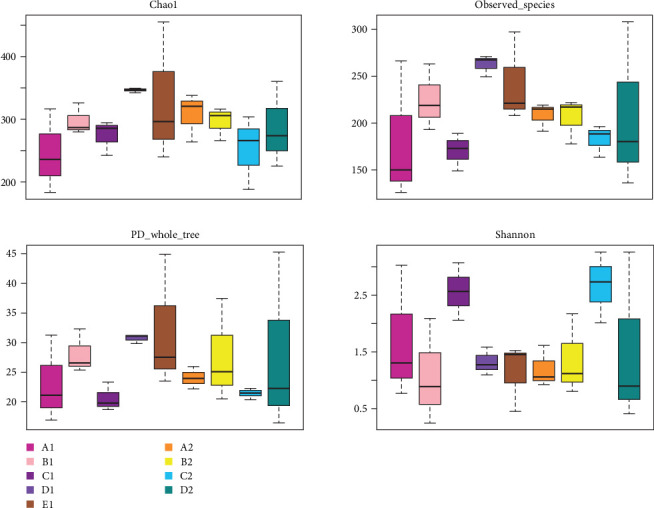
Alpha diversity index of *H. crassispina* fed on different diets. A1–E1 are intestinal walls of feeding corn, carrot, sweet potato, kelp, and nonfeeding group, respectively, A2–D2 are intestinal contents of feeding corn, carrot, sweet potato, and kelp, respectively. The box chart contains five data nodes arranged from largest to smallest, namely maximum, upper quartile, median, lower quartile, and minimum.

There were significant differences in biodiversity indicators of five diet groups (*p* < 0.05). The box plots of biodiversity indices for each group are shown in Figure 7. In intestinal wall of the purple sea urchin, the median values for the Chao1, observed_species, and PD_whole_tree indices were highest in D1, indicating that kelp positively affected the species richness and phylogenetic diversity of intestinal wall microbiota. In intestinal contents, the median values for the Chao1, observed_species, and PD_whole_tree indices were higher in A2, suggesting an enhancement in microbial species richness. The Shannon index revealed that C1 and C2 exhibited the highest median values in both intestinal wall and contents, indicating that sweet potatoes favored the evenness and diversity of intestinal microbiome. Additionally, extreme values were observed in D2 and E1, suggesting anomalously high systematic diversity in individual samples fed with kelp or in a state of starvation (Figure 7).

#### 3.3.3. Beta Diversity Analysis

**Figure 8:**
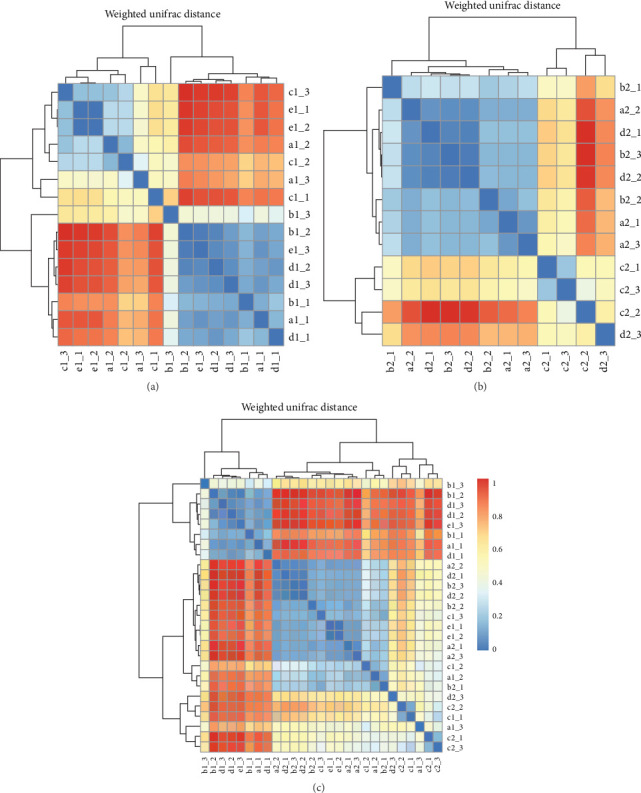
Beta diversity indices of microbial community differences among *H. crassispina* fed on different diets. In the figure, (A) represents the differences in microbial communities among purple sea urchin intestinal wall samples fed with different diets; (B) represents the intestinal content samples, and (C) is the aggregate of all samples. In a single chart, the letters represent different feeding groups: “a” for corn-fed group, “b” for carrot-fed group, “c” for sweet potato fed group, “d” for kelp-fed group, and “e” for nonfed group. The numbers 1 and 2 represent the intestinal wall and intestinal contents, respectively. The suffixes _1 to _3 indicate three replicates.

Within intestinal wall, carrot and kelp groups displayed a high degree of internal similarity (indicated by the deeper blue blocks in the heatmap), suggesting substantial similarity in species abundance within these groups. In contrast, corn and sweet potato groups showed greater distances between samples, indicating significant variation in species abundance within these groups. The clustering tree (part of the dendrogram) indicated a higher variability in the microbial communities within intestinal wall of no feeding group (Figure 8A). All sea urchins fed with different diets demonstrated a higher similarity of microbial communities within intestinal content (Figure 8B). However, significant microbial community differences were observed between the intestinal wall and intestinal content in both carrot and kelp groups, whereas corn and sweet potato groups showed more similarity between the microbial communities of intestinal wall and intestinal content (Figure 8C).

#### 3.3.4. Analysis of Microbial Species Composition in Fed Different Diets From *H. crassispina*

**Figure 9:**
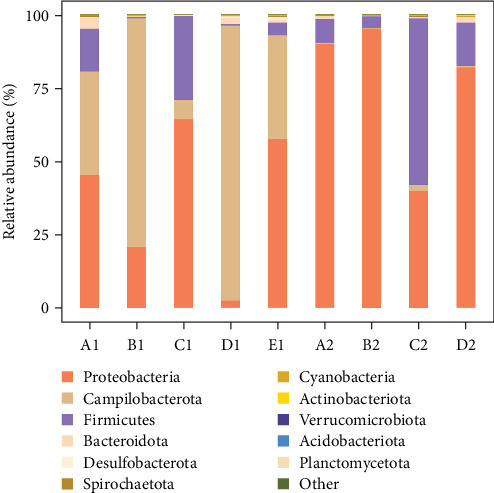
Distribution map of microorganisms in *H. crassispina* fed with different diets at phylum level. A1–E1 are intestinal walls of feeding corn, carrot, sweet potato, kelp, and nonfeeding group, respectively, A2–D2 are intestinal contents of feeding corn, carrot, sweet potato, and kelp, respectively.

At the phylum level, microbial analysis revealed 32 phyla in intestinal wall and 24 phyla in the contents of purple sea urchins. The dominant bacterial phyla were Proteobacteria (55.34%), Campilobacterota (27.99%), and Firmicutes (14.77%). Additionally, other phyla such as Bacteroidota (1.1%), Desulfobacterota (0.25%), and Spirochaetota (0.19%) exhibited higher abundance in specific groups: Bacteroidota in corn intestinal wall (Group A1), Desulfobacterota in kelp-fed intestinal wall (Group D1), and Spirochaetota in no feeding intestinal wall (Group E1), as well as in corn contents (Group A2) and kelp-fed contents (Group D2). Notably, the abundance of Proteobacteria in kelp-fed intestinal wall (Group D1) was significantly lower than in other groups, with a higher proportion of Campilobacterota. Proteobacteria showed a significantly lower abundance in intestinal wall compared to the contents, while Campilobacterota displayed the opposite trend. The abundance of Proteobacteria in the contents from Groups A2–D2 was significantly higher than in intestinal wall from Groups A1–E1 (*p* < 0.05) (Figure 9).

**Figure 10:**
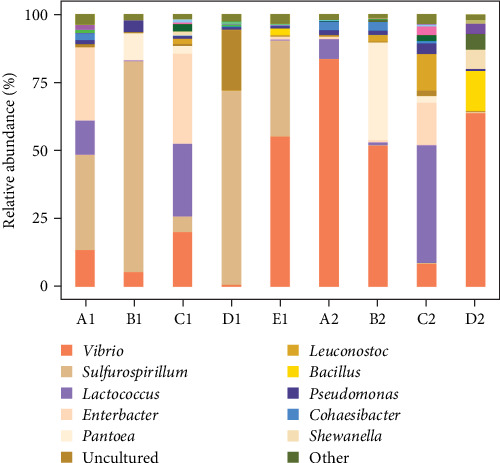
Distribution map of microorganisms in *H. crassispina* fed with different diets at genus level. A1–E1 are intestinal walls of feeding corn, carrot, sweet potato, kelp, and nonfeeding group, respectively, A2–D2 are intestinal contents of feeding corn, carrot, sweet potato, and kelp, respectively.

At the genus level, a total of 330 bacterial genera were identified in intestinal wall samples of the purple sea urchin, while 283 genera were detected in intestinal contents, with 21 of the top 50 abundant genera being identified (others were categorized as “unclassified” or “norank”). The dominant genera were *Vibrio* (33.57%), followed by *Sulfurospirillum* (25.06%), *Lactococcus* (10.15%), *Enterobacter* (8.72%), and *Pantoea* (5.7%). Genera with less than 5% abundance included *Leuconostoc*, *Bacillus*, *Pseudomonas*, and *Cohaesibacter*, with all other genera comprising less than 1% of the total. Specifically, *Vibrio* had the highest relative abundance in no feeding group, while the lowest was observed in kelp group. Carrot group showed a higher proportion of *Sulfurospirillum*, and *Lactococcus* was more abundant in corn group and sweet potato group compared to others. In the analysis of intestinal contents, *Vibrio* also had the highest relative abundance, with *Lactococcus* being the most dominant in sweet potato group, followed by *Vibrio*. Additionally, *Enterobacter* showed relatively high abundance in Groups A1 and C1, while *Lactococcus* was notably abundant in Groups A1, C1, A2, and C2 (Figure 10).

## 4. Discussion

### 4.1. Impact of Different Diets on the Gonadal Development of *H. crassispina*

Diets serve as crucial nutritional sources for the growth and development of sea urchins [33, 34]. Kelp is considered the ideal natural feed for sea urchins [35, 36]. However, the fresh supply of kelp is mainly available from December to May, leading to a 7-month supply gap. Therefore, it is necessary to explore effective alternative diets available throughout the year to ensure stable food supply and improve gonadal color and texture. Although alternatives such as Irish moss (*Gracilaria*) and sea lettuce (*Ulva lactuca*) have been attempted in previous studies, their effectiveness is limited [8, 37, 38]. Thus, this study aims to investigate the impact of year-round available natural diets on the gonadal development of purple sea urchins, with the goal of identifying superior alternatives for gonadal maturation promotion. The experiment revealed that diets such as corn, sweet potatoes, and carrots could promote sea urchin growth, but their effectiveness was inferior to that of kelp. Previous studies have demonstrated that kelp is the most effective feed for promoting the growth of sea urchins, as evidenced by an increase in body weight and test diameter [39, 40]. This finding is consistent with the results of the present study.

This study found that the purple sea urchin-fed corn exhibited the highest GSI, while there was no significant difference between the kelp group and the two other natural feed groups. This is consistent with the findings of Sartori et al. [22], that corn can serve as an alternative feed to algae and significantly increase the gonadal index of sea urchins when combined with spinach. Basuyaux and Blin [21] had previously discovered a synergistic effect between corn and algae, which could enhance food conversion rates without affecting sea urchin growth [21]. Corn primarily consists of starch, with protein accounting for only 10% of its dry weight [41]. The effective absorption of starch varies among species, with Lawrence [42] noting that certain species of sea urchins could absorb polysaccharides as efficiently as soluble proteins [42]. The results of this study further demonstrate the significant advantages of feeding corn alone, reinforcing the feasibility of using corn as an alternative or complementary feed of algae to achieve better maturation promotion effects. Robinson and Colborne [43] found that green sea urchins fed with cabbage and carrots had superior gonadal development compared to those fed with large algae [43]. In contrast, our study found no significant difference in gonadal indices among the groups fed with carrots, sweet potatoes, and kelp, suggesting that their effects may be more pronounced in other aspects rather than accelerating gonadal maturation.

Gonadal color is a key factor determining consumer acceptance [44]. In recent years, many studies have aimed to improve gonad flavor by feeding artificial diets, finding that high-quality gonads are generally orange to yellow in color, while those fed with artificial diets tend to have lighter gonadal colors [45]. Gonadal color is mainly determined by the carotenoids in the gonads, most of which are synthesized from dietary precursors [7, 46]. Our study showed that no feeding group exhibited the greatest differences in gonadal color, suggesting that the lack of diets might lead to deviations from the ideal gonadal color. In contrast, the gonadal color sea urchin in corn group was closer to orange-yellow. Ghaeni, Roomiani, and Moradi [47] demonstrated that zeaxanthin, a primary carotenoid, was found in spirulina, corn, and kelp. Corn and its products are also considered major sources of zeaxanthin and lutein in the diet [48]. Additionally, Carboni et al. [49] showed that kelp was rich in *β*-carotene, lutein, and zeaxanthin [49]. The superiority of gonadal color in kelp group in our study further confirms this point. The redness (a^*∗*^) values of carrot and sweet potato groups (23.18 ± 5.54, 24.27 ± 3.47) were close to standard yellow (28.7) and orange-red, but the lightness (L^*∗*^) values were low, and the total color differences (*Δ*E_1_ and *Δ*E_2_) were higher than those in corn group. Carrots are rich in *β*-carotene [25], and sweet potatoes are also one of the richest sources of *β*-carotene [50], while lower levels of zeaxanthin in corn contribute to its lower yellowness values. In conclusion, kelp as the main feed source has a positive effect on the growth and development of sea urchins, while corn has a better effect on the gonad index and color.

### 4.2. Effects of Different Diets on the Nutrient Composition of *H. crassispina*

#### 4.2.1. Impact on Amino Acid Composition

This study analyzed the influence of different diets on the amino acid composition of purple sea urchin gonads. A comparison of amino acid content revealed that kelp-fed group exhibited the highest levels of TAAs, EAAs, and NEAAs. This indicates that feeding kelp provides a rich and efficient source of amino acids, particularly EAAs. The EAA/TAA ratio, an important indicator of protein nutritional value, is ideally 0.4 [51]. In this study, the EAA/TAA ratio was 0.385 in the kelp-fed group, 0.33 in the corn group, 0.30 in the carrot group, 0.28 in the sweet potato group, and 0.31 in the no feeding group, close to the ideal value, significantly higher than that of the other groups.

Sea urchins cultivated in kelp beds exhibit advantages over those inhabiting exposed rocky areas in terms of color, texture, and taste [52–55]. Additionally, research by Takagi et al. [55] suggested that wild sea urchins harvested during periods of kelp scarcity exhibited significantly poorer taste compared to those artificially fed [55]. In this study, kelp-fed group demonstrated the highest umami amino acid content among all groups, with glutamic acid being particularly prominent. Glutamic acid not only contributes to enhancing the taste of gonads but also possesses antioxidant properties, promoting oxidative reactions [56]. This underscores the potential of kelp as a feed to improve gonad taste. However, the high content of TBAAs (such as arginine) in gonads might negatively affect taste [13]. Nevertheless, the abundant free amino acids in kelp [57] still exert a positive effect on gonad taste. This contradictory phenomenon reveals the complex and subtle influence of kelp on enhancing gonad taste.

It is notable that the corn group exhibited higher levels of sweet amino acids compared to other groups, particularly in terms of alanine and proline content. It has been discovered for the first time that feeds other than kelp can increase the sweet amino acid levels in sea urchins. The comprehensive comparison reveals that although carrot group and sweet potato group have slight advantages in influencing the taste of the sea urchin gonads, their overall effects are far from comparable to those of kelp and corn.

#### 4.2.2. Impact on Fatty Acid Composition

This study found that among all SFAs, myristic acid (C14:0) and palmitic acid (C16:0) were the most abundant, consistent with the findings of Rincón-Cervera et al. [58] in Chilean sea urchin gonads [58]. Similarly, the main fatty acids of white sea urchin in Taiwan's natural environments are C14:0, C16:0, and C20:0 [59]. Additionally, EPA (C20:5n3), as the most abundant PUFA, was found to be higher in both kelp-fed and no feeding groups. It is known for its beneficial effects on cardiovascular health [60], anti-inflammatory properties, and brain development, and is a key fatty acid for promoting larval growth [61]. Higher levels of *α*-linolenic acid were found in corn, kelp, and sweet potato groups, while the highest levels of eicosatrienoic acid and ARA were found in kelp group. Notably, linoleic acid (C18:2n6), which is essential for the growth of sea urchins [88], was highest in corn group. Sea urchins could synthesize ARA and EPA using *α*-linolenic acid and linoleic acid as precursors [62–64]. However, ARA, an eicosanoic acid precursor [65], could interfere with normal growth when ingested in excess by sea urchins [62]. n-3 and n-6 PUFAs are essential for heart health, brain development, and cellular growth [66], but excessive ingestion might have side effects [67, 68]. n-3/n-6 PUFA ratio was highest in kelp group, which was similar to the results of Shao et al.'s [69] study, whereas corn group had a relatively low level of n-3 PUFA, which might be due to the effect of its own high linoleic acid content [70]. The composition of sea urchin gonads is influenced by diet type, with significant effects observed on fatty acid synthesis and composition [71]. This effect is not limited to adult sea urchins, but might also affect the lipid composition of juveniles [61].

The results on fatty acid composition indicate that kelp is an excellent feed for sea urchins, enhancing the nutritional value of their fatty acid profile. Corn can also be appropriately combined to increase the total fatty acid content. These findings provide guidance for the selection of sea urchin feed and the improvement of lipid nutritional quality in aquaculture.

### 4.3. Effects of Different Diets on the Intestinal Microbiome of *H. crassispina*

Intestinal microbes play a pivotal role in the metabolism, transport, and signaling of substances in the host. They are capable of metabolizing and translocating a variety of carbohydrates, which could have a profound effect on the health of the host [72, 73]. This study revealed significant differences in the intestinal microbial composition of sea urchins under different fed various diets. Kelp group exhibited the highest median of Chao1, Observed_species, and PD_whole_tree indices, as well as the highest weighted UniFrac distance, indicating a higher diversity and richness of microbial communities. The high fucoidan content in kelp provided sufficient energy for intestinal microbes, promoting their diversity and richness. In contrast, corn group exhibited lower intestinal microbial diversity than the other groups. This might be attributed to the easily digestible carbohydrate properties of corn, which prompted the anabolic phylum to participate in the decomposition of macromolecular organic matter, including proteins and polysaccharides [74]. Second, corn and sweet potato groups exhibited higher median Shannon's indexes in the intestinal wall and contents, suggesting that corn and the sweet potato might contribute to the promotion of microbial community homogeneity and diversity. The extreme values observed in kelp (D2) and no feeding (E1) groups might indicate that higher phylogenetic diversity in individual samples under kelp feeding or starvation was possibly due to differences in microbial composition of individuals from different genetic backgrounds [75]. The clustering tree demonstrates greater variability of microbial communities in the intestinal wall no feeding group, which suggests that environmental conditions and intrinsic variation of organisms play an important role in the formation of microbial community structure. This might reflect the fact that microbes can colonize in intestinal tract of sea urchin according to their own environment, particularly in the absence of external food input.

Campylobacter phylum was previously classified as part of the Ascomycetes, which is a core member of the sea urchin intestinal microbiota [76, 77]. The phylum Ascomycota is a highly diverse bacterial phylum encompassing numerous species that are critical to the health of aquatic ecosystems. Members of this phylum are capable of rapid reproduction and possess a variety of functions, including decomposition of organic matter, nitrification, and denitrification [78]. The phylum Bacteroidetes, which is present in food particles of purple sea urchin, is essential for digestion in sea urchins. This group of microorganisms contains enzymes that could break down carbohydrates, which helps sea urchins to compensate for their deficiencies in digestive enzymes and significantly facilitates the digestion and absorption of sea urchins, especially in the processing of indigestible substances such as complex polysaccharides [74, 77]. This finding was also confirmed in studies of echinoderm sea cucumbers. Furthermore, the abundance of the Anabaena phylum was positively correlated with the level of the ARA content in the dietary group of sea cucumbers [79]. In our study, ARA was higher in kelp group (group D1) than in corn group (group A1) and sweet potato group (group C1). Consequently, the abundance of the anamorphic phylum was higher. These bacteria promote the intestine absorption of energy from food and the production of compounds associated with fat deposition [79]. It was demonstrated that the specific dominant groups present in kelp could metabolize high levels of ARA and also adjust the abundance of the thick-walled bacterial phylum.

*Vibrio* of phylum Mycobacterium was found to be significantly more abundant in sea urchin intestinal food particles than in the intestinal wall. This is consistent with the results of Hakim for *Vibrio* [75]. The results suggest that *Vibrio* might play a key role in the metabolism of proteins and lipids and might be involved in the biosynthesis of lipids and fatty acids in sea urchins [80, 81]. We found that the abundance of *Vibrio* on the intestinal wall in no feeding group was significantly higher than in the intestinal walls of the other diet groups, particularly the abundance of some pathogenic bacteria such as *Vibrio harveyi* [82, 83]. This might be the reason for the continuous spine loss and poor mobility observed in sea urchins under starvation. However, this finding is inconsistent with the conclusion that *Vibrio* is beneficial for the synthesis of lipids and fatty acids in sea urchins. Further research is needed to identify the *Vibrio* species that is advantageous to sea urchins. Furthermore, the rapid passage of food through the intestine might not be conducive to microbial colonization of intestine, when food is plentiful [84]. Additionally, the dominant genus, *Thiospirillum*, exhibited a higher abundance in the study. It was observed that sea urchins in starvation conditions could nibble that can be captured, such as silt and sediment. This might be attributed to the ability of the sea urchins to break down indigestible materials in intestine. The higher abundance of carrots and kelp in the intestinal wall of sea urchins fed on carrots and kelp might be due to the presence of absorption-inhibiting fibers in carrots and sulfates in algae [85, 86]. These substances help break down sulfur-containing organic matter and thus inhibit harmful *Vibrio* by elevating the colonization of *Helicobacter*. Furthermore, our study demonstrated that the abundance of *Lactobacillus* in the intestines of sea urchins fed with corn and sweet potato baits significantly increased. This indicates that *Lactobacillus* can colonize in intestines and form a dominant flora, which is also one of the factors that inhibit the abundance of harmful *Vibrio*. Consequently, high-starch baits such as corn and sweet potato can be utilized to cultivate beneficial intestinal microbiota. The colonization of sea urchins with *Lactobacillus* might facilitate the efficient utilization of feed, resulting in enhanced growth rates and overall health. This discovery offers novel feed strategies for the sea urchin aquaculture industry. The production of beneficial biomass by these flora not only enhances host immunomodulation but also improves intestinal structure and promotes metabolism and growth [87].

## 5. Conclusion

This study analyzed the impact of different diets on the gonadal development of *H. crassispina*. The results confirmed that besides kelp, corn significantly enhances gonadal growth, and has potential for nutritional complementarity in combined diet formulations. Kelp and corn were particularly notable for their amino acid and fatty acid profiles, with high levels of umami and sweet amino acids, respectively, enhancing the taste quality of the gonads. Fatty acid component analysis showed high contents of EPA, ARA, linolenic acid, and linoleic acid in both kelp and corn can improve the fatty acid composition of sea urchins. Diet choice significantly affected the microbial diversity in the intestinal of purple sea urchins; kelp increased microbial community diversity, while corn and sweet potato diets increased the abundance of lactobacillus in the intestinal, highlighting their important role in maintaining intestinal health. Future work should focus on optimizing feed formulations to balance EAAs, fatty acids, and other nutrients to further enhance the nutritional value of the gonads. Promoting high-starch content diets like corn and sweet potatoes could benefit the intestinal microbial community of sea urchins, particularly by increasing lactobacillus, positively impacting the digestive capacity and overall health of sea urchins. Therefore, further research should explore the deeper mechanisms of interaction between diet and sea urchin intestinal microbiota, providing more effective nutritional management strategies for the healthy rearing of sea urchins.

## Figures and Tables

**Table 1:** Effects of different diets on growth and development of *H. crassispina*.

Items	M1 (kelp)	M2 (corn)	M3 (carrot)	M4 (sweet potatoes)	M5 (no feeding)
IBW (g)	51.26 ± 26.33	57.11 ± 20.62	49.27 ± 12.79	54.29 ± 17.2	58.8 ± 24.37
FBW (g)	56.67 ± 26.35	58.94 ± 21.02	51.22 ± 12.94	56.77 ± 15.85	59.13 ± 24.3
WGR (%)	5.41 ± 1.00^a^	1.84 ± 1.19^bc^	1.95 ± 1.01^bc^	2.49 ± 2.41^b^	0.33 ± 2.34^c^
SDGI (%)	0.32 ± 0.09^a^	0.22 ± 0.07^ab^	0.19 ± 0.05^ab^	0.17 ± 0.05^ab^	0.11 ± 0.04^ab^
SHGI (%)	0.16 ± 0.06^a^	0.13 ± 0.04^ab^	0.10 ± 0.02^ab^	0.09 ± 0.03^ab^	0.07 ± 0.02^ab^
GSI (%)	2.47 ± 1.05^b^	6.459 ± 2.31^a^	1.863 ± 0.85^bc^	2.49 ± 0.93^b^	0.77 ± 0.32^c^

*Note*: Values with superscript indicate significant differences between different groups in the intergroup at *p* < 0.05.

Abbreviations: FBW, final body weight; GSI, gonadosomatic index; IBW, initial body weight; SDGI, shell diameter growth rate; SHGI, shell high growth rate; WGR, weight gain rate.

**Table 2:** Effects of different diets on gonad color of *H. crassispina*.

Items	M1 (kelp)	M2 (corn)	M3 (carrot)	M4 (sweet potatoes)	M5 (no feeding)
L*⁣*^*∗*^	46.95 ± 9.79^a^	47.43 ± 7.46^a^	41.34 ± 9.1^ab^	38.6 ± 10.82^b^	34.32 ± 6.68^b^
a*⁣*^*∗*^	15.58 ± 4.29^b^	13.97 ± 2.49^b^	23.18 ± 5.45^a^	24.27 ± 3.47^a^	14.07 ± 2.26^b^
b*⁣*^*∗*^	31.85 ± 9.92^ab^	36.58 ± 4.57^a^	29.3 ± 6.82^ab^	23.53 ± 10.48^b^	24.93 ± 7^b^
*Δ*E1	38.71 ± 13.46^b^	35.38 ± 7.16^b^	43.15 ± 10.72^ab^	37.2 ± 14.23^b^	51.73 ± 9.48^a^
*Δ*E2	46.24 ± 13.48^ab^	41.74 ± 7.38^b^	51.05 ± 10.78^ab^	57.84 ± 14.44^a^	59.49 ± 9.52^a^

*Note*: Values with superscript indicate significant differences between different groups in the intergroup at *p* < 0.05. *Δ*E_1_: difference value between gonad color and orange-yellow; *Δ*E_2_: between gonad color and yellow.

**Table 3:** Amino acid composition of gonads of *H. crassispina* fed with different diets (WM basis) (g/100 g).

Amino acid	M1 (kelp)	M2 (corn)	M3 (carrot)	M4 (sweet potatoes)	M5 (no feeding)
Aspartic acid	0.76 ± 0.22^a^	0.417 ± 0.07^b^	0.43 ± 0.03^b^	0.41 ± 0.08^b^	0.59 ± 0.31^a^
Glutamic acid	1.06 ± 0.26^a^	0.58 ± 0.19^b^	0.52 ± 0.04^b^	0.47 ± 0.11^b^	0.55 ± 0.33^b^
∑TSAA	1.82 ± 0.48^a^	0.99 ± 0.26^bc^	0.95 ± 0.07^bc^	0.88 ± 0.19^bc^	1.14 ± 0.64^b^
Threonine	0.40 ± 0.12^bc^	0.78 ± 0.06^a^	0.17 ± 0.02^c^	0.46 ± 0.04^b^	0.147 ± 0.15^c^
Serine	0.33 ± 0.08^ab^	0.23 ± 0.04^b^	0.18 ± 0.02^bc^	0.393 ± 0.031^a^	0.19 ± 0.13^bc^
Glycine	0.63 ± 0.06^b^	0.92 ± 0.09^a^	0.60 ± 0.02^b^	0.567 ± 0.076^b^	0.35 ± 0.13^c^
Alanine	0.33 ± 0.06^a^	0.35 ± 0.04^a^	0.33 ± 0.02^a^	0.353 ± 0.032^a^	0.38 ± 0.095^a^
Proline	0.19 ± 0.05^bc^	0.63 ± 0.05^a^	0.113 ± 0.01^c^	0.32 ± 0.02^b^	0.24 ± 0.07^bc^
∑TUAA	1.89 ± 0.37^bc^	2.91 ± 0.27^a^	1.40 ± 0.08^c^	2.09 ± 0.21^b^	1.31 ± 0.58^c^
Cysteine	0.10 ± 0.03^b^	0.05 ± 0.02^c^	0.06 ± 0.02^c^	0.048 ± 0.01^c^	0.14 ± 0.03^a^
Valine	0.51 ± 0.13^a^	0.19 ± 0.07^b^	0.16 ± 0.02^bc^	0.15 ± 0.04^bv^	0.13 ± 0.16^bc^
Methionine	0.20 ± 0.07^b^	0.06 ± 0.03^c^	0.06 ± 0.01^c^	0.60 ± 0.02^a^	0.21 ± 0.11^b^
Isoleucine	0.36 ± 0.09^a^	0.14 ± 0.05^bc^	0.14 ± 0.01^bc^	0.13 ± 0.04^bc^	0.18 ± 0.13^b^
Leucine	0.55 ± 0.13^a^	0.28 ± 0.10^b^	0.21 ± 0.02^bc^	0.20 ± 0.05^bc^	0.19 ± 0.20^bc^
Tyrosine	0.40 ± 0.10^a^	0.19 ± 0.05^c^	0.153 ± 0.01^c^	0.153 ± 0.02^c^	0.28 ± 0.09^b^
Phenylalanine	0.37 ± 0.10^a^	0.15 ± 0.05^bc^	0.14 ± 0.02^bc^	0.14 ± 0.03^bc^	0.20 ± 0.13^b^
Lysine	0.64 ± 0.20^a^	0.21 ± 0.07^abc^	0.27 ± 0.02^bc^	0.24 ± 0.07^abc^	0.37 ± 0.31^b^
Histidine	0.16 ± 0.03^a^	0.11 ± 0.04^ab^	0.07 ± 0.01^b^	0.07 ± 0.02^b^	0.18 ± 0.06^a^
Arginine	0.82 ± 0.30^a^	0.29 ± 0.147^b^	0.25 ± 0.02^b^	0.20 ± 0.06^bc^	0.14 ± 0.33^c^
∑TBAA	4.11 ± 1.16^a^	1.67 ± 0.60^bc^	1.51 ± 0.15^bc^	1.87 ± 0.35^b^	2.02 ± 1.53^b^
TAA	7.82 ± 1.91^a^	5.58 ± 1.06^b^	3.86 ± 0.21^c^	4.84 ± 0.57^bc^	4.48 ± 2.70^bc^
EAA	3.00 ± 0.79^a^	1.86 ± 0.42^b^	1.16 ± 0.10^bc^	1.38 ± 0.29^bc^	1.39 ± 1.14^bc^
NEAA	4.82 ± 1.24^a^	3.72 ± 0.72^b^	2.70 ± 0.20^c^	3.46 ± 0.46^b^	3.08 ± 1.61^c^

*Note:* Values with superscript indicate significant differences between different groups in the intergroup at *p* < 0.05.

Abbreviations: EAA, essential amino acid; NEAA, nonessential amino acid; TAA, total amino acid; TSAA, tasty amino acid.

**Table 4:** Composition of gonadal fatty acids in *H. crassispina* fed with different diets (WM basis) (μg/g).

Fatty acid	M1 (kelp)	M2 (corn)	M3 (carrot)	M4 (sweet potatoes)	M5 (no feeding)
C14:0	3386.18 ± 1691.07^bc^	5225.97 ± 1217.25^ab^	4027.16 ± 1280.57^abc^	6672.56 ± 1031.49^a^	1999.07 ± 1648.24^c^
C15:0	65.40 ± 41.92^a^	71.88 ± 54.2^a^	47.484 ± 28.72^a^	73.07 ± 26.823^a^	86.17 ± 49.87^a^
C16:0	2901.76 ± 1392.49^a^	2684.75 ± 1503.24^a^	1908.59 ± 1366.14^a^	3361.7 ± 789.69^a^	1978.29 ± 1339.52^a^
C18:0	300.06 ± 107.5^a^	196.49 ± 100.51^a^	130.38 ± 77.64^a^	190.61 ± 59.16^a^	228.28 ± 90.37^a^
C20:0	69.93 ± 23.72^a^	36.29 ± 16.09^b^	25.83 ± 20.28^b^	35.06 ± 13.58^b^	42.57 ± 9.61^ab^
C22:0	8.788 ± 1.35^a^	9.089 ± 2.45^a^	8.067 ± 0.56^a^	9.908 ± 1.19^a^	10.88 ± 1.76^a^
∑SFA	6732.12 ± 3258.1^b^	8224.47 ± 2893.74^a^	6147.51 ± 2773.9^b^	10342.9 ± 1921.9^a^	4345.26 ± 3139.3^c^
C14:1	80.67 ± 43.61^b^	592.67 ± 137.79^a^	239.11 ± 62.91^b^	765.72 ± 217.36^a^	60.57 ± 43.01^b^
C15:1	1.07 ± 0.18^a^	1.265 ± 0.31^a^	1.11 ± 0.21^a^	1.374 ± 0.205^a^	1.245 ± 0.171^a^
C16:1	163.25 ± 64.01^c^	350.33 ± 131.11^ab^	219.23 ± 83.11^bc^	502.01 ± 124.01^a^	101.21 ± 41.39^c^
C18:1N12T	48.62 ± 12.26^a^	70.11 ± 28.712^a^	47.61 ± 18.21^a^	83.88 ± 21.88^a^	45.91 ± 23.68^a^
C18:1N12	25.69 ± 8.82^a^	28.23 ± 7.55^a^	7.18 ± 2.55^b^	9.543 ± 3.187^b^	6.91 ± 1.34^b^
C18:1N9C	156.63 ± 46.45^b^	343.7 ± 97.72^a^	58.81 ± 45.14^b^	85.32 ± 18.12^b^	55.28 ± 17.014^b^
C18:1N7	393.26 ± 184.03^a^	567.95 ± 294.56^a^	381.28 ± 247.78^a^	727.49 ± 206.15^a^	307.81 ± 198.59^a^
C19:1N12T	16.35 ± 7.45^a^	21.95 ± 14.38^a^	16.87 ± 1.38^a^	26.69 ± 8.34^a^	27.54 ± 9.68^a^
C19:1N9T	18.96 ± 6.95^b^	22.57 ± 10.92^b^	23.23 ± 13.47^b^	47.09 ± 8.46^a^	13.64 ± 8.54^b^
C20:1T	128.08 ± 38.24^a^	138.68 ± 76.57^a^	103.61 ± 67.98^a^	159.85 ± 66.02^a^	155.421 ± 67.209^a^
C20:1	564.61 ± 228.96^a^	717.16 ± 264.74^a^	363.25 ± 382.84^a^	517.33 ± 215.66^a^	286.69 ± 153.46^a^
C22:1N9	331.07 ± 130.42^a^	293.45 ± 190.78^a^	177.51 ± 114.97^a^	274.01 ± 110.94^a^	325.25 ± 195.99^a^
∑MUFA	1928.30 ± 771.42^b^	3148.07 ± 1255.14^ab^	1638.81 ± 1040.53^bc^	3200.31 ± 1000.33^a^	1387.48 ± 760.08^c^
C18:3N3	48.36 ± 15.70^a^	49.83 ± 16.08^a^	30.07 ± 6.53^ab^	43.557 ± 5.61^a^	13.92 ± 2.12^b^
C20:3N3	64.90 ± 17.76^a^	41.19 ± 24.11^ab^	27.33 ± 13.11^b^	48.034 ± 17.78^ab^	22.51 ± 7.63^b^
C20:5N3 (EPA)	676.24 ± 251.00^a^	280.31 ± 268.29^bc^	121.63 ± 35.52^c^	221.312 ± 49.875^bc^	594.05 ± 263.69^ab^
C22:5N3	19.96 ± 8.71^ab^	17.67 ± 12.04^ab^	5.987 ± 4.303^b^	15.201 ± 6.428^ab^	25.945 ± 13.571^a^
C22:6N3 (DHA)	38.15 ± 24.63^ab^	38.42 ± 13.91^ab^	7.55 ± 1.94^b^	50.32 ± 30.96^a^	54.53 ± 19.74^a^
∑n-3 PUFA	847.63 ± 317.81^a^	427.44 ± 334.43^b^	192.57 ± 61.39^c^	378.42 ± 110.64^b^	710.95 ± 306.75^ab^
C18:2N6	100.79 ± 40.43^bc^	792.86 ± 169.82^a^	221.59 ± 53.91^b^	121.18 ± 14.77^bc^	55.19 ± 41.244^c^
C18:3N6	71.30 ± 19.75^a^	8.95 ± 3.95^b^	5.985 ± 6.47^b^	7.833 ± 1.841^b^	11.46 ± 4.37^b^
C20:3N6	77.11 ± 25.04^b^	131.2 ± 31.38^a^	57.544 ± 3.76^bc^	53.59 ± 2.42^bc^	32.94 ± 16.98^c^
C20:4N6 (ARA)	893.42 ± 252.24^a^	496.29 ± 239.39^b^	294.36 ± 54.66^b^	365.44 ± 38.01^b^	617.64 ± 186.28^ab^
C22:5N6	12.74 ± 5.08^a^	12.65 ± 6.37^a^	4.69 ± 1.65^a^	16.16 ± 11.03^a^	16.51 ± 6.155^a^
∑n-6 PUFA	1155.38 ± 342.56^b^	1441.94 ± 450.91^a^	584.18 ± 120.44^d^	564.21 ± 68.07^d^	733.74 ± 255.03^c^
n-3/n-6 PUFA	0.73 ± 0.18^b^	0.3 ± 0.08^c^	0.32 ± 0.08^c^	0.67 ± 0.14^bc^	0.97 ± 0.38^a^

*Note:* Values with superscript indicate significant differences between different groups in the intergroup at *p* < 0.05.

Abbreviations: ARA, arachidonic acid; DHA, docosahexaenoic acid; EPA, eicosapentaenoic acid; MUFA, monounsaturated fatty acid; PUFA, polyunsaturated fatty acid; SFA, saturated fatty acid.

## Data Availability

The data that support the findings of this study are available from the corresponding author upon reasonable request.
